# Computational discovery of conserved RNA structures and functional characterization of a structured lncRNA in *Leishmania braziliensis*

**DOI:** 10.1016/j.ncrna.2025.05.010

**Published:** 2025-05-20

**Authors:** Caroline R. Espada, Christian Anthon, Rubens D.M. Magalhães, José Carlos Quilles Junior, Natalia M.M. Teles, Fabiano S. Pais, Lissur A. Orsine, Letícia de Almeida, Tânia P.A. Defina, Adam Dowle, Jan Gorodkin, Pegine B. Walrad, Angela K. Cruz

**Affiliations:** a Department of Cell and Molecular Biology, Ribeirão Preto Medical School, University of São Paulo FMRP-USP, Ribeirão Preto, São Paulo, Brazil; b Center for Non-coding RNA in Technology and Health, Department of Animal and Veterinary Sciences, Faculty for Health and Medical Sciences, University of Copenhagen, Copenhagen, Denmark; c Department of Biology, York Biomedical Research Institute, University of York, York, United Kingdom; d Data Science Lab, Metabolomics and Proteomics Lab, Bioscience Technology Facility, Department of Biology, University of York, York, United Kingdom

**Keywords:** Non-coding RNAs, *Leishmania braziliensis*, RNA structure, Gene expression regulation, CRISPR- Cas9

## Abstract

*Leishmania* parasites alternate between hosts, facing environmental changes that demand rapid gene expression adaptation. Lacking canonical RNA polymerase II promoters, transcription in these eukaryotes is polycistronic, with gene regulation occurring post-transcriptionally. Although non-coding RNAs (ncRNAs) have been identified in *Leishmania* transcriptomes, their functions remain unclear. Recognizing RNA structure's importance, we performed a genome-wide alignment of *L. braziliensis* and related species, identifying conserved RNA structures, 38 of which overlap with known ncRNAs. One such ncRNA, *lncRNA45*, was functionally characterized. Using a knockout cell line, we demonstrated that *lncRNA45* is crucial for parasite fitness. Reintroducing the wild type *lncRNA45* restored fitness, while a version with a single nucleotide substitution in the structured region did not. This mutation also altered RNA-protein interactions. These findings suggest that *lncRNA45*'s regulatory role and protein interactions rely on its secondary structure. This study highlights the significance of structured lncRNAs in *Leishmania* biology and their potential as therapeutic targets. Further research into these ncRNAs could uncover new parasite regulation mechanisms and inspire novel treatment strategies.

## 1 Introduction

*Leishmania* is a protozoan parasite that causes a group of diseases collectively known as leishmaniasis [1]. Approximately 20 species are pathogenic to humans, causing a range of clinical syndromes from skin lesions to potentially fatal visceral leishmaniasis [1]. *Leishmania* (*Viannia*) *braziliensis* is among the most clinically significant pathogenic species in South America, causing mucocutaneous leishmaniasis—a severe form of the disease that can lead to extensive destruction of mucosal tissues [2]. Currently, there are no vaccines for humans, and available treatments often have severe side effects as well as decreasing efficacy due to parasite resistance [2]. To complete its life cycle, the heteroxenous *Leishmania* must adapt to significant environmental changes [3]. During a blood meal on an infected mammalian host, female Phlebotomine sandflies ingest phagocytes containing intracellular amastigotes (AMA). Within the insect gut, these amastigotes differentiate into procyclic promastigotes (PRO). After replication, PRO migrate to the anterior gut and further differentiate into intermediate forms until they reach the infective stage—metacyclic promastigotes (META). During the subsequent blood meal, META are regurgitated into the dermis of a vertebrate host, where they are phagocytized by macrophages and differentiate back into AMA [3]. Therefore, rapid and precise regulation of gene expression is essential for efficient transmission and, consequently, parasite survival.

Unlike other eukaryotes, trypanosomatids such as *Leishmania* exhibit a unique genetic organization, lacking canonical RNA polymerase II (RNA Pol II) promoters for individual genes [4]. Instead, transcription start sites (TSS) drive tandem arrays of genes, resulting in polycistronic transcription in which functionally unrelated genes within the same polycistronic transcriptional units (PTUs) are transcribed together as a single polycistronic mRNA [4]. Each PTU may contain over 200 genes, all of which undergo *trans*-splicing during RNA processing. In this process, a 39-nucleotide capped exon (spliced leader RNA, SL-RNA) is added to the 5′-end of each mRNA, and the preceding mRNA is polyadenylated at its 3′-end [5]. Mature mRNAs are then exported to the cytoplasm for translation into proteins. Despite this distinctive genetic organization, the mechanisms governing gene expression regulation are not fully understood. Post-transcriptional regulation is crucial involving mRNA processing, stability, nuclear-cytoplasmic transport, and translation [4,6]. RNA-binding proteins (RBPs) are pivotal in recognizing specific sequences or structural motifs in mRNA untranslated regions (UTRs), influencing their stability, localization, or translation [7].

Non-coding RNAs (ncRNAs) were shown to act as gene expression regulators in trypanosomatids [8,9]. They are transcribed but not translated into proteins and are generally classified as either short (<200 bp, sncRNAs) or long (>200 bp, lncRNAs) [10]. This classification, however, is currently under revision [11]. Despite the large number of ncRNAs identified in the transcriptomes of trypanosomatids [8,9,[12], [13], [14]], their biological functions and biogenesis remain incompletely understood. In *L. braziliensis*, 11,372 putative ncRNAs (3602 of them being differentially expressed across lifecycle stages) have been identified through RNA-seq and comparative transcriptomics suggesting they could be enrolled in gene expression regulation in this parasite [13].

Many ncRNAs, such as tRNAs, rRNAs and miRNAs, are strongly structured and function through structure. LncRNAs appear not too strongly structured but may contain structural regions from which they function [15]. In addition, some lncRNAs were shown to function through structural elements rather than conserved nucleotide sequences, highlighting the importance of secondary structures [16]. Based on the importance of secondary structure for ncRNAs' role in living systems, using a computational approach we searched for conserved secondary structures of ncRNAs in trypanosomatid genomes [17], particularly in *L. braziliensis* MHOM/BR75/M2903, and identified ncRNAs with conserved secondary structures differentially expressed between morphologies, suggesting their biological relevance. Based on differential expression and genomic localization, we selected a lncRNA with a conserved secondary structure for further investigation into its potential regulatory function. We found that this lncRNA is involved in parasite growth in culture, likely through interactions with proteins implicated in gene expression regulation and cell-cell signaling pathways. Moreover, we found that a single base substitution that alters its secondary structure impairs its function in *L. braziliensis*. Overall, elucidating the role of ncRNAs—and particularly their structural dynamics—provides important insights into the regulatory mechanisms essential for the survival and transmission of *Leishmania* parasites.

## 2 Results

### 2.1 Assembly of trypanosomatid genomes for comparative analysis

In this study, we selected eleven trypanosomatids genomes to search for RNA conserved secondary structures. We observed that, for most genomes, the percentage of complete Euglenozoa orthologues (from a total of 130) determined by the BUSCO methodology was above 90 %. This confirms the quality of genome assembly and annotation, except for the genomes of *Bodo saltans* and *Trypanosoma cruzi* (Table 1). Although these genomes exhibit lower conservation levels, *Bodo saltans* and *Trypanosoma cruzi* were included in the analysis to ensure representation of diverse evolutionary lineages; their percentages of complete orthologue remained higher than those of fragmented or missing sequences. The percentage of complete orthologues (C (%), Table 1) exceeded the percentage of fragmented (F (%), Table 1) or missing sequences (M (%), Table 1). We aligned these eleven genomes against the *Leishmania braziliensis* MHOM/BR75/M2903 genome using Lastz [18]. The resulting pairwise alignments were merged into a *L. braziliensis*-based multiple alignment using MultiZ [19] and the UCSC tool chain (see Methods for details). Details of genome quality, including percentages of complete, fragmented, and missing orthologues, are presented in Table 1.

**Table 1:** BUSCO analysis of trypanosomatids’ genomes. The universal orthologues used for this analysis were obtained from the BUSCO euglenozoan Odb10 dataset.

Species^a^	BUSCO (euglenozoa – 130)^b^			
	**C (%)**	**CD (%)**	**F (%)**	**M (%)**
*Leishmania major* Friedlin	98.5	0	0	1.5
*Leishmania donovani* BPK282A1	97.7	0	0	2.3
*Leishmania infantum* JPCM5	97.7	0	0	2.3
*Leishmania amazonensis* MHOM/BR/1973/M2269	96.9	0	0.8	2.3
*Leishmania panamensis* MHOM/COL/81/L13	96.9	0	1.5	1.6
*Crithidia fasciculata* CF-C1	96.2	0	2.3	1.5
*Leishmania braziliensis* MHOM/BR/75/2903	93.8	0	4.6	1.6
*Trypanosoma brucei* TREU927	93.1	7.7	3.1	3.8
*Bodo saltans* LakeKonstanz	72.3	0.8	9.2	18.5
*Trypanosoma cruzi* CL Brener Non-Esmeraldo-like	66.9	0	3.8	29.3
*Trypanosoma cruzi* CL Brener Esmeraldo-like	54.6	0	3.1	42.3

a Species of Trypanosomatids (genome files from TriTrypDB v44).

b BUSCO analysis results: (Complete - C (%)), percentage of 130 Euglenozoa universal single copy orthologues identified as full sequence orthologues in the listed genomes. (Complete Duplicated - CD (%)) full sequence found in more than one position in the genome. (Fragmented – F (%)) only partial sequence was found or; (Missing – M(%)) sequence not found.

### 2.2 Prediction of conserved structures

To predict conserved RNA structures in the *L. braziliensis* genome, we used MultiZ/roast to generate a multiple alignment of 10 *Leishmania* species genomes, covering 99.3 % of the *L. braziliensis* genome. From the multiple alignment we constructed 12,969 alignment windows that overlap with the lncRNA annotation. On these windows, we predicted conserved RNA structures using RNAz [20]. RNAz yielded 631 ncRNA candidates with a support vector machine (SVM) score ≥0, the threshold for classifying a window as having a conserved RNA structure.

Infernal (version 1.1.2) [21] in conjunction with Rfam (version 14.1) [22] predicted 177 known structured RNAs in the *L. braziliensis* genome. A comparison between the 177 known structures and the RNAz predictions indicated that 226 of the 12,969 alignment windows overlapped with 64 of the known structures by at least 40 bases. We analyzed the 12,969 alignment windows in several ways and determined that the average pairwise sequence identity and the number of organisms/sequences in each alignment window were the most efficient filters (Supplementary Fig. 1 and Supplementary Fig. 2). We filtered the alignment windows by requiring an average pairwise sequence identity ≥70 % and at least 6 organisms in the alignment (if 7 or more valid sequences were present, RNAzWindow automatically selected the 6 best sequences based on optimal pairwise sequence identity). After this filter, we are left with 6035 alignment windows, of which 206 overlap known RNA structures.

**Fig. 1:**
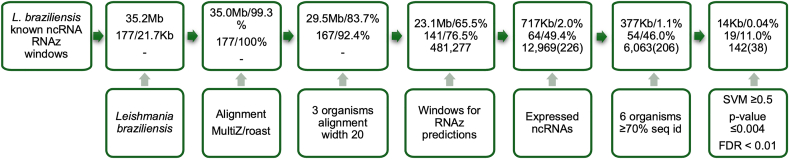
Filtering of multiple alignments (99.3 % genome coverage) yielded 12,969 windows, predicting 142 structured RNAs (104 novel, 38 overlapping 177 known RNAs from Infernal/Rfam). Bottom panels show genome coverage, expressed ncRNA coverage, and structure predictions.

To estimate the false discovery rate (FDR) for the RNAz predictions, we shuffled the 6035 alignment windows 100 times each while preserving di-nucleotide composition. The SVM scores from these shuffled windows provided a background distribution from which p-values for the RNAz predictions were derived (Supplementary Fig. 3). Based on the p-values of the 206 windows overlapping known ncRNAs, we selected an SVM score cut-off of 0.5, which corresponds to a p-value ≤0.004 (Supplementary Figs. 3 and 4). To avoid GC bias for individual candidates, we used the 100 shuffled windows for each candidate as its own background, requiring that all shuffled windows exhibit SVM scores lower than that of the candidate. This ensures that the per-candidate FDR is <0.01. Applying the filtering criteria (background p-value ≤0.004 and per candidate FDR <0.01) yielded a final set of 142 candidates, of which 104 are novel and 38 overlap known ncRNAs. In addition, we recovered 19 out of 54 expressed, highly conserved ncRNAs (38 out of 206 windows, see Fig. 1).

In the final selection, we focused on candidates located between two CDSs on the same strand, which is true for a good portion of the known ncRNAs (∼55 % within 10,000 and ∼38 % within 5000 bases). We found that 74 of our 104 novel candidates are located between two CDSs (≤5000). The ncRNAs differentially expressed in *L. braziliensis* morphologies, were chosen to be functionally analyzed. This choice was based on the possibility that these ncRNA play a role in parasite differentiation being essential for life cycle progression and parasite survival, providing novel insights into the mechanisms orchestrating parasite-host interactions.

### 2.3 Predicted point mutations that can affect the structures of target ncRNAs

Considering its coverage, size, genomic location, and differential expression, the long non-coding RNA *LbrM2906_26_lncRNA45* (hereafter *lncRNA45*) was selected as a candidate for functional characterization. This lncRNA contains a conserved structured region, referred to as Locus 709.

**Fig. 2:**
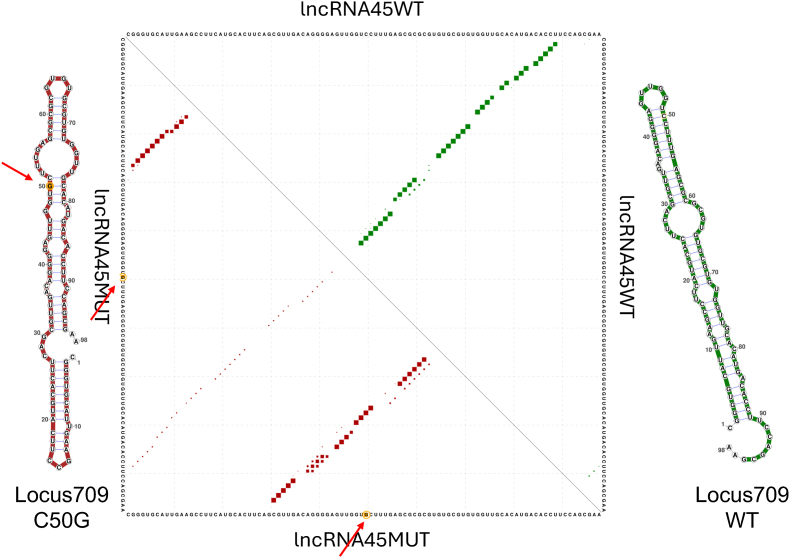
RNAsnp analysis of a C-to-G mutation at position 50: comparison between base-pair probabilities of *lncRNA45*MUT (dark red) and *lncRNA4*5WT (green); MFE structure of *lncRNA45*MUT (left – dark red) and *lncRNA45* (WT) MFE structure (right – green), and G-to-A mutation at position 76 (bottom right). Red arrows and orange circles indicate the C50G substitution.

To assess potential mutations affecting RNA secondary structure, stability, or interactions with other macromolecules, *in silico* predictions were performed using the RNAsnp tool. Among several potential mutations, the cytosine-to-guanine substitution at position 50 (C50G) within Locus 709 was predicted to significantly disrupt the secondary structure of *lncRNA45* (p-value = 0.004). Interestingly, although many other single nucleotide polymorphisms (SNPs) resulted in structures markedly different from the wild-type —such as the guanine-to-adenine substitution at position 76 (G76A) (Fig. 2)—it is possible that the functional conformation corresponds to the G76A variant. If so, the C50G mutation might destabilize this structure. However, confirming this hypothesis would require further investigation beyond the scope of the present study.

### 2.4 *lncRNA45* is processed and is predominantly localized to the cytoplasm of *L. braziliensis*

*lncRNA45* was identified in the *L. braziliensis* M2903 transcriptome and predicted to be a non-coding RNA (ncRNA) by two predictors—ptRNApred (classified as RNaseP family) and RNAcon (classified as Intron-gp-1) [13]. The predicted size of *lncRNA45* is 408 nt, it is located in an intergenic region on chromosome 26 (coordinates: 296,265–296,672) on the minus strand, the same strand sense of the PTU it belongs to (Supplementary Fig. 5A) [13]. *LncRNA45* is situated between two coding sequences (CDSs): an upstream gene encoding a putative NMD3 family protein (LbrM2903_260014600.1), and a downstream gene coding for a hypothetical protein (LbrM2903_260014500.1) [23] (Supplementary Fig. 5B). The RNA coverage profile strongly suggests that *lncRNA45* is not part of the untranslated regions (UTRs) of either coding sequence (CDS) (Supplementary Fig. 5B). In addition, the RNA-seq coverage profile indicates that *lncRNA45* is preferentially expressed in amastigotes (Supplementary Fig. 5A and 5B), showing a 1.53-fold higher abundance in amastigotes compared to other morphological forms. However, RT-qPCR analysis did not confirm the RNA-seq results, showing instead that *lncRNA45* is more abundant in promastigotes (PRO) than in other morphological forms (Supplementary Fig. 5C).

To confirm the size of *lncRNA45* and to investigate its processing following polycistronic transcription, we employed an RNA circularization-based protocol [24] (Supplementary Fig. 5D). Total RNA from *L. braziliensis* M2903 was decapped (TAP^+^) or not (TAP^−^) using tobacco acidic phosphatase (TAP), circularized, and reverse transcribed using target-specific primers. Primers directed towards the 5′ and 3’ ends were used to amplify a product containing the transcript ends and its modifications (poly(A) tail and spliced leader, if present). A distinct and specific band was observed in both TAP^+^ and TAP^−^ reactions (Supplementary Fig. 5E). These bands were cloned into pGEM-T and sequenced. Fifteen clones (ten from TAP^+^ and five from TAP^−^ reactions) were collected, amplified with M13-F and M13-R primers, and sequenced. Nine clones presenting different fragment sizes after amplification were sequenced and mapped to the locus of *lncRNA45* on *L. braziliensis* M2903 chromosome 26.

**Fig. 3:**
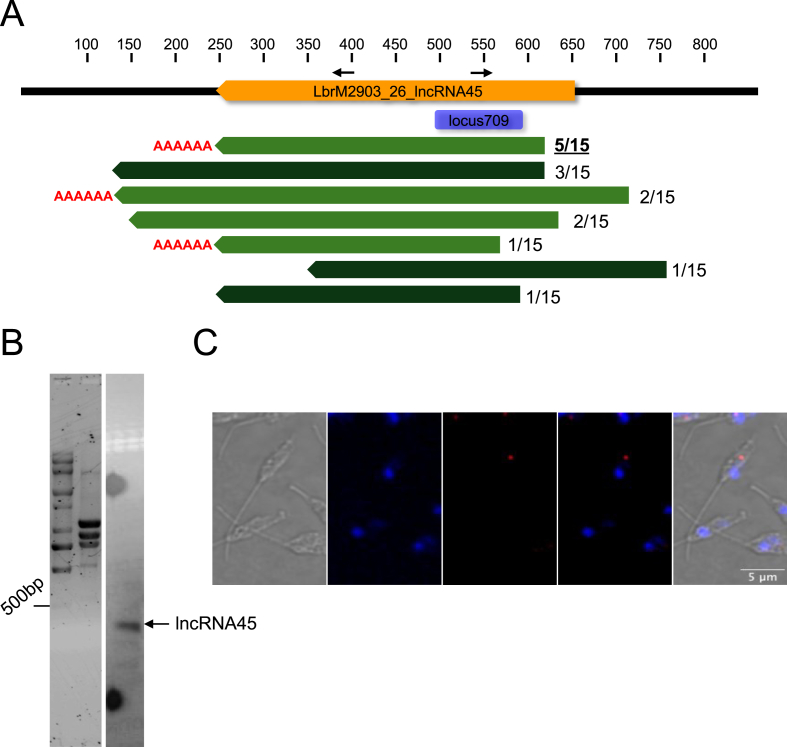
*LncRNA45* is a 407-nt, structured and polyadenylated long noncoding RNA, observed in the cytoplasm of *Leishmania braziliensis*. **(A)** RNA circularization assay confirms polyadenylation and transcript processing. Total RNA was treated with (TAP+, light green) or without (TAP−, dark green) tobacco acid pyrophosphatase, circularized, reverse-transcribed, PCR-amplified, and sequenced. Among 15 sequenced clones, 7 distinct transcript sizes were identified. Poly(A) tails were found in 8 out of 15 clones (3 transcript profiles), all derived from TAP + RNA. The most frequently observed transcript profile is highlighted (bold and underlined). Positioning of the structured locus 709 is depicted in purple. Orange bar indicates the computationally predicted ncRNA45. Slim black arrows above the orange bar indicates positioning of primers used in the circularization assay. **(B)** Northern blot analysis detected a single transcript <500 nt corresponding to *lncRNA45* in total RNA from promastigotes. White lines observed in the upper portion of the blot are photographic artifacts. Sybr Green staining shows RNA marker and rRNA bands as loading control. **(C)** RNA FISH reveals *lncRNA45* (red), is in the cytoplasm of *L. braziliensis.* Parasites' nuclei were stained with DAPI (blue). Images acquired at 100 × magnification using a Zeiss multiphoton microscope.

While *in silico* predictions suggested that *lncRNA45* is 407 nucleotides long, the circularization assay revealed seven distinct transcript profiles ranging from 325 to 585 bp (Fig. 3A). Among these, four cDNA clones lacked a poly(A) tail in the 3′ UTR, whereas three clones possessed a poly(A) tail consisting of 14–17 adenines (Fig. 3A). No spliced leader sequence was observed in the 5’ UTR of *lncRNA45*, suggesting that its capping may occur via a mechanism distinct from spliced leader-mediated capping. Interestingly, three different transcript sizes lacking polyadenylation were identified in the TAP- reactions, whereas three out of four clones sequenced from the TAP + reactions were polyadenylated. The most abundant transcript in TAP + samples (5 out of 10 clones) was 376 nucleotides long and had a 13-nucleotide poly(A) tail (indicated by the black arrow in Fig. 3A), corresponding to the region of highest coverage observed for *lncRNA45* (Fig. 3A). Notably, except for *lncRNA45*_TAP+_Clone1, all other identified *lncRNA45* isoforms contain the conserved structured Locus 709 identified in comparative analysis.

The existence of *lncRNA45* as an independent transcript in *L. braziliensis* M2903 total RNA extract was also confirmed by northern blotting using sequence-specific radiolabeled probes (Fig. 3B). Northern blotting revealed a specific, unique band smaller than 500 bp for *lncRNA45*, confirming the presence of this ncRNA in *L. braziliensis* and consistent with its expected size of 408 nucleotides.

The localization of *lncRNA45* was determined using RNA fluorescence in situ hybridization (FISH) with probes designated to bind along its entire length. FISH results revealed that *lncRNA45* was predominantly observed in the cytoplasm of *L. braziliensis* (Fig. 3C and Supplementary Fig. 6). In some cells, multiple fluorescence signals were observed; however, due to experimental limitations, we could not determine whether each signal corresponded to a single *lncRNA45* molecule or to multiple copies.

### 2.5 Knockout of *lncRNA45* results in reduced parasite density in culture

To investigate the biological function of *lncRNA45*, *L. braziliensis* M2903 cell lines either knocked out (Δ*lncRNA45*) for *lncRNA45* or overexpressing (OE_*lncRNA45*) this transcript, were generated. Using CRISPR/Cas9, null mutants lacking *lncRNA45* were successfully generated, and the complete loss of *lncRNA45* was confirmed by PCR (Supplementary Fig. 7A and Supplementary Fig. 7E – PCR A). *L. braziliensis* M2903 overexpressing *lncRNA45* (OE_*lncRNA45*) was obtained by transfecting the parasites with the pSSU-*lncRNA45*-Sat plasmid, followed by selection with nourseothricin (Supplementary Fig. 7B and Supplementary Fig. 7F – PCR B). As a mock control, parasites were transfected with the pSSU-GFP-Sat plasmid (OE_mock) (Supplementary Fig. 7C and Supplementary Fig. 7F – PCR C).

**Fig. 4:**
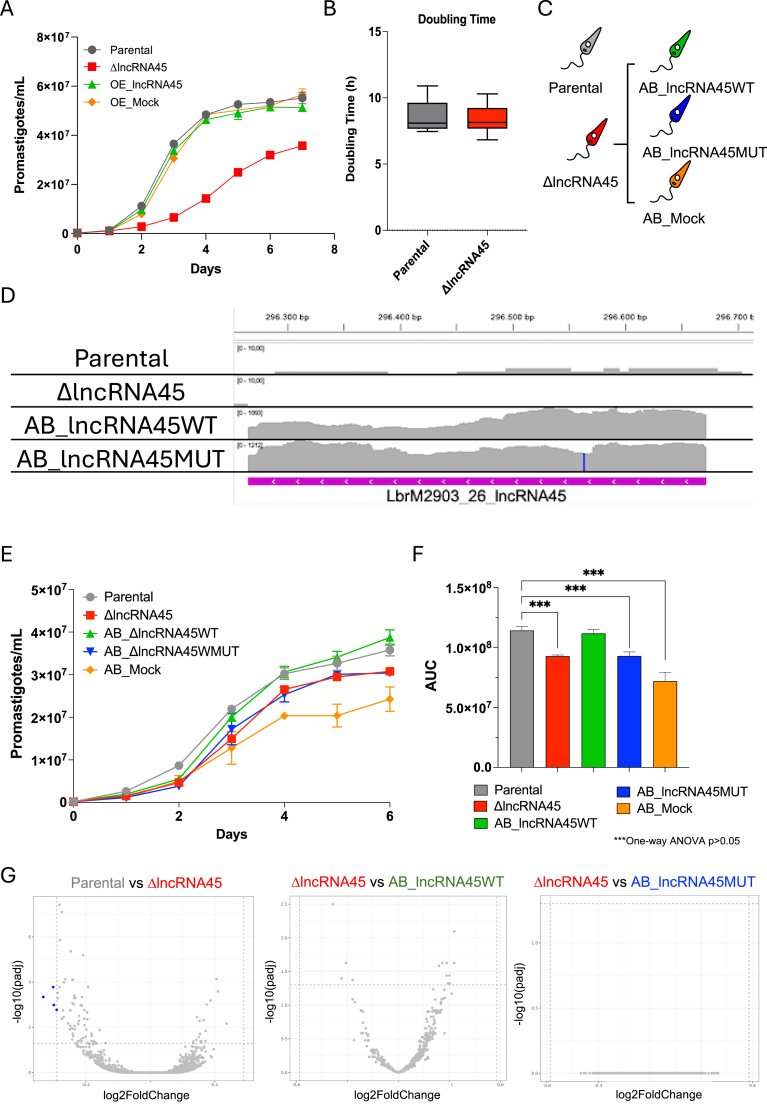
Impact of *lncRNA45* knockout on *L. braziliensis* fitness. **(A)** Growth curves of *L. braziliensis* M2903 OE_lncRNA45 (green), OE_Mock (orange), and parental cells (gray). ΔlncRNA45 cells exhibited reduced growth compared to the parental line. The assay was performed with three biological replicates, and similar results were obtained in at least three independent experiments. Due to the low variability among replicates, a representative experiment is shown. Area Under the Curve (AUC) analysis was performed using one-way ANOVA followed by Tukey's test (p < 0.05). **(B)** Doubling time of Δ*lncRNA45* vs. parental cells (non-parametric *t*-test, *p* < 0.05). **(C)** Δ*lncRNA45* was complemented with *lncRNA4*5WT (green) sequence, the sequence carrying the C50G mutation (*lncRNA45*MUT - blue), and the GFP-coding gene (AB-Mock - orange) using pSSU-SAT plasmid. **(D)** IGV visualization of transcriptomic data from Parental, ΔlncRNA45, AB_lncRNA45WT, and AB_lncRNA45MUT cell lines. Integrated Genomics Viewer (IGV) tracks show normalized RNA-seq read coverage (gray pileups) across the *lncRNA45* genomic region. The ΔlncRNA45 track displays a complete absence of reads, confirming successful knockout of *lncRNA45*. In contrast, both AB_lncRNA45WT and AB_lncRNA45MUT show restored expression of the transcript. The blue vertical line over RNA-reads map of AB_lncRNA45MUT represents the position of the substituted nucleotide. The pink bar corresponds to the annotation of lncRNA45. **(E)** Growth curves of *L. braziliensis* ΔlncRNA45 and add-back cell lines over a 7-day period. The ΔlncRNA45 line consistently exhibited reduced growth compared to the parental strain. **(F)** Quantification of growth by area under the curve (AUC) analysis. Statistical significance was assessed using one-way ANOVA followed by Tukey's post hoc test (*p* < 0.05). Data represent the mean of three biological replicates. **(G)** Transcriptomic analysis of *lncRNA45* knockout and add-back cell lines. Volcano plots of differential expression (DESeq2, p < 0.05, FC > 1.5x) analysis between Parental vs. knockout (Δ*lncRNA45*), knockout vs. add-back complemented with the WT sequence of *lncRNA45* (AB_*lncRNA4*5WT), and knockout vs. add-back complemented with the *lncRNA45* sequence containing a C50G substitution at Locus 709 (AB_*lncRNA45*C50G). Blue dots represent genes with significant differential expression, while gray dots indicate non-significant differences.

To investigate whether parasite fitness was impaired, these transfectants (Δ*lncRNA45*, OE_*lncRNA45*, and OE_mock) along with the parental cell line, were subjected to assays mimicking key steps of the *Leishmania* life cycle. Promastigote growth was assessed by adjusting the culture to 2 × 10^5^ promastigotes/mL and counting cells for at least 7 days or until reaching the stationary phase. Area under the curve analysis showed significantly reduced growth for Δ*lncRNA45*, with a cell density of 3.6 × 10^7^ promastigotes/mL on the 7th day of culture, compared to 5.5 × 10^7^ promastigotes/mL in the parental cell line (Fig. 4A). No significant differences were observed for OE_*lncRNA45* or OE_mock, compared to the parental cell line (One-way ANOVA and Tukey's multiple-comparison test, p < 0.05).

To determine if the reduced cell density observed after 7 days for the Δ*lncRNA45* cell line was due to reduced replication capacity, doubling time was measured by adjusting the culture to 1 × 10^6^ promastigotes/mL and counting after 24 h for 4 days. No significant differences in doubling time were observed for Δ*lncRNA45* compared to the parental cell line (Fig. 4B) (non-parametric *t*-test, p < 0.05).

When inside the vector, procyclic promastigotes migrate to the anterior portion of the phlebotomine gut and face an environment with reduced nutrient availability. The ability to withstand nutritional stress was evaluated for Δ*lncRNA45* and OE_*lncRNA4*5 by incubating these cells in PBS for 4 h, followed by recovery in M199 for 24 h. The percentage of viable cells after stress was determined relative to non-stressed cells and compared to the parental line. No significant differences were observed in the recovery capacity from nutritional stress for any of the transfectant lines compared to the parental cell line (Supplementary Fig. 8A) (One-way ANOVA and Tukey's multiple-comparison test, p < 0.05).

To infect the mammalian host, procyclic promastigotes need to differentiate into infective metacyclic promastigotes. Since procyclic and metacyclic promastigotes differ in size and density, using a Ficoll gradient, we determined the percentage of metacyclic promastigotes in relation to procyclic forms for Δ*lncRNA45*, OE_*lncRNA45*, as well as the parental and OE_mock cell lines. The percentages of metacyclic promastigotes in culture (% of metacyclogenesis) observed for the parental, Δ*lncRNA45*, OE_*lncRNA45*, and OE_mock cell lines were 5.7 % ± 1.7 (mean ± SEM), 6.3 % ± 1.5, 5.3 % ± 1.9, and 5.3 % ± 0.6, respectively, with no significant differences observed between the cell lines (Supplementary Fig. 8B) (One-way ANOVA and Tukey's multiple-comparison test, p < 0.05).

The capacity to infect THP-1-derived macrophages and the replication of intracellular amastigotes were also assessed for Δ*lncRNA45*, OE_*lncRNA45*, and OE_mock in comparison to the parental cell line but no significant differences were observed in the percentage of infected macrophages (Supplementary Fig. 8C) or in the number of amastigotes per macrophage (Supplementary Fig. 8D) were observed up to 96 h post-infection for any transfectant compared to the parental cell line (One-way ANOVA and Tukey's multiple-comparison test, p < 0.05).

Once inside the macrophages, *Leishmania* parasites are exposed to reactive oxygen species, which they must survive to replicate and establish infection. The ability to recover from oxidative stress induced by hydrogen peroxide (H_2_O_2_) was evaluated for Δ*lncRNA45*, OE_*lncRNA45*, and OE_mock in comparison to the parental cell line. The percentage of recovery was determined in comparison to non-stressed parasites, and no significant differences were observed for the transfectant lines compared to the parental cell line (Supplementary Fig. 8E).

### 2.6 Complementation of Δ*lncRNA45* with *lncRNA4*5WT restores promastigote growth

To investigate whether the biological function of *lncRNA45* is related to the conserved secondary structure at Locus709 the wild-type (*lncRNA4*5WT) and mutant (*lncRNA45*MUT, carrying the C50G substitution at Locus 709) sequences of *lncRNA45* were cloned into the pSSU-Sat plasmid, the same vector used to overexpress this lncRNA in OE_*lncRNA45* transfectants. After cloning, the plasmids were sequenced to ensure C50G was the only SNP present in the sequence and transfected into the *L. braziliensis* M2903 Δ*lncRNA45* cell line. The plasmid carrying the GFP gene instead of the *lncRNA45* sequence (pSSU-Mock) was also transfected into Δ*lncRNA45* as a control. Thus, three clonal lines were generated over the *L. braziliensis* M2903 Δ*lncRNA45* background (Fig. 4C).

The absence of endogenous *lncRNA45* was confirmed by PCR in all the transfectants (Supplementary Fig. 7G - PCR A). The presence of the plasmid containing *lncRNA4*5WT or *lncRNA45*MUT was confirmed in the add-back cell lines AB_*lncRNA4*5WT and AB_*lncRNA45*MUT, respectively (Supplementary Fig. 7G - PCR B). The presence of the plasmid pSSU-GFP was confirmed in the AB_Mock cell line (Supplementary Fig. 7G - PCR C). Amplification of a 0.32 kb fragment of *hsp70* CDS was used as control for DNA presence and integrity (Supplementary Fig. 7G - PCR D). RNA-seq data analysis also confirmed the absence of reads mapping to *lncRNA45* in the Δ*lncRNA45* cell line, proving that this lncRNA was indeed deleted (Fig. 4D). The RNA-seq data further confirmed that expression of *lncRNA45* was re-established in the add-back and that C50G is the only nucleotide substitution in the *lncRNA45*MUT sequence (Fig. 4D – blue line).

A 7-day growth curve was generated, as described previously. As observed before, reduced growth was seen in Δ*lncRNA45* compared to the parental cell line (Fig. 4A and 4E–F). The add-back of the *lncRNA45*WT sequence restored the growth of Δ*lncRNA45* to the levels of the parental cell line (Fig. 4E–F). Supporting the significance of Locus 709 and requirement of *lncRNA45*, neither the *lncRNA45*MUT nor mock add-back control (AB_Mock) could restore Δ*lncRNA45* growth to the levels of the parental line (Fig. 4E–F).

### 2.7 *lncRNA45* does not regulate other mRNA levels

To investigate if *lncRNA45* regulates the abundance of other transcripts in *L. braziliensis*, RNA-seq was conducted for the parental, Δ*lncRNA45*, AB_*lncRNA4*5WT, and AB_*lncRNA45*MUT cell lines. Only two genes were found to be differentially expressed in the knockout (Δ*lncRNA45*) compared to the parental cell line (DESeq2 p < 0.05, FC > 1.5) (Fig. 4G). No significant differences in gene expression were observed between the knockout and add-back cell lines, suggesting that *lncRNA45* does not regulate global mRNA levels in *L. braziliensis*.

### 2.8 *lncRNA45*-protein interaction is modified in the presence of C50G substitution in locus 709

The fact that *lncRNA45* is differentially expressed between morphologies and has a conserved structured locus together with the fact that its deletion from *L. braziliensis* genome impacts promastigote fitness, strengthened the hypothesis that this lncRNA might be involved in gene expression regulation in this parasite. To investigate which pathways *lncRNA4*5 might regulate, an *in vitro* pulldown assay was conducted to identify the proteins interacting with this lncRNA.

The pulldown was performed using both the wild-type sequence of *lncRNA45* (*lncRNA4*5WT) and a sequence containing the C50G substitution (*lncRNA45*MUT) aiming to determine if the RNA-protein interaction is dependent on the secondary structure formed by Locus 709. The *lncRNA4*5WT and *lncRNA45*MUT sequences were fused to a 4xS1m aptamer and sequenced to ensure that C50G was the only divergence between the sequences. Since S1m has an affinity for streptavidin-coated beads (SA-beads), *lncRNA45* was immobilized and exposed to log-phase *L. braziliensis* M2903 promastigote protein lysate. As a control, an S1m sequence without the *lncRNA45* fusion was used.

**Fig. 5:**
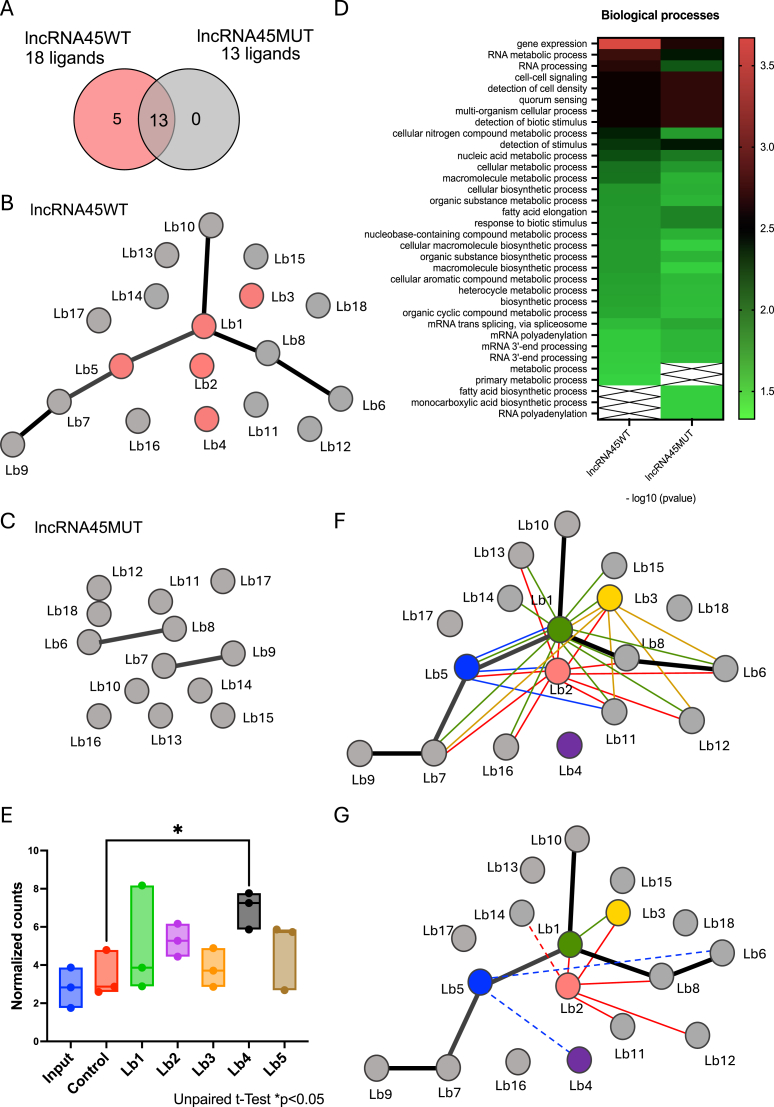
In vitro pulldown of *lncRNA4*5WT and *lncRNA45*MUT sequences. **(A)** Comparison of proteins binding to *lncRNA4*5WT (18 proteins) and *lncRNA45*MUT (13 proteins), highlighting five lost interactions due to the C50G substitution at locus 709. Thirteen proteins bind to both variants. **(B, C)** Protein-protein interaction networks for *lncRNA4*5WT **(B)** and *lncRNA45*MUT **(C)** determined using String. The interactive networks can be accessed for *lncRNA4*5WT (https://www.ndexbio.org/#/network/0175a6c3-274c-11ef-9621-005056ae23aa?accesskey=e4fe291b38d4e92ee1b1585d11e0ca5bf624fdf6e669a3155c711dde39d39089) and for *lncRNA45*MUT (https://www.ndexbio.org/#/network/d3df8921-274a-11ef-9621-005056ae23aa?accesskey=e505803df77aa75dbf287c55f80c2226fb71bfc82ac437ab93cd47d4975ec1db). **(D)** Heat map of gene ontology (GO) enrichment analysis of binding proteins. Terms are classified by significance (*p* < 0.05). **(E)** RIP analysis confirms *lncRNA45*-protein interaction, with significant enrichment in Lb4 eluate (*p* < 0.05). **(F)** Network analysis of predicted and RIP-identified *lncRNA45* binders. Circles represent proteins identified in the S1m assay (Lb1-Lb18), with colored circles indicating RIP-validated interactions. **(G)** Comparison of proteins in RIP eluates with and without RNase treatment. Dotted lines indicate indirect RNA-dependent interactions absent in negative control comparisons.

Proteins recovered after filter selection and significantly enriched in all three replicates of *lncRNA4*5WT and *lncRNA45*MUT compared to the empty control filter were considered binding partners of these sequences. A total of 18 and 13 proteins were identified as binders for *lncRNA4*5WT and *lncRNA45*MUT, respectively (Table 2, Fig. 5A). Interestingly, the 13 proteins recovered from the *lncRNA45*MUT pulldown were common to the *lncRNA4*5WT pulldown (Table 2). The other five proteins were lost in the presence of the C50G substitution in the Locus 709 sequence of *lncRNA45* (*lncRNA45*MUT) (Table 2, Fig. 5A).

**Table 2:** *lncRNA45*-protein interactions observed in S1m *in vitro* pull-down.

lncRNA45						
Code	Description^a^	Accession Number^b^	GeneID M2904^c^	GeneID M2903^c^	Size^d^	pValue^e^
**Lb1**	**40S ribosomal protein S9, putative**	**A4H519**	**LbrM.07.0750**	**LBRM2903_070014500**	**22 kDa**	**95 % (0.0029)**
**Lb2**	**XRN 5′-3′ exonuclease N-terminus, putative**	**A4H8G4**	**LbrM.16.0400**	**LBRM2903_160009500**	**108 kDa**	**95 % (0.011)**
**Lb3**	**Lupus La protein homolog, putative**	**A4HBQ1**	**LbrM.21.0600**	**LBRM2903_210010600**	**37 kDa**	**95 % (0.029)**
**Lb4**	**hypothetical protein, conserved**	**A4HGJ6**	**LbrM.28.1600**	**LBRM2903_280022100**	**80 kDa**	**95 % (0.014)**
**Lb5**	**Nop53 (60S ribosomal biogenesis), putative**	**A4HMS1**	**LbrM.34.2420**	**LBRM2903_340031800**	**38 kDa**	**95 % (0.0080)**
Lb6	RNA-binding protein, putative	A4HI65	LbrM.30.1230	LBRM2903_300017800	67 kDa	95 % (0.014)
Lb7	tRNA (Uracil-5-)-methyltransferase, putative	E9AIL9	LbrM.20.5870	LBRM2903_200073900	66 kDa	95 % (0.047)
Lb8	DNA-directed RNA polymerase II subunit 3, putative	A4HH22	LbrM.29.0150	LBRM2903_290006800	37 kDa	95 % (0.037)
Lb9	dihydrouridine synthase (Dus), putative	A4H8X8	LbrM.17.0330	LBRM2903_170009100	66 kDa	95 % (0.037)
Lb10	Mitochondrial small ribosomal subunit Rsm22, putative	A4HD89	LbrM.24.0330	LBRM2903_240008300	110 kDa	95 % (0.031)
Lb11	ISWI complex protein	A4HEU2	LbrM.26.0880	LBRM2903_260013600	80 kDa	95 % (0.026)
Lb12	kinetoplast-associated protein, putative	A4HQA1	LbrM.35.6130	LBRM2903_350072700	14 kDa	95 % (0.041)
Lb13	acetyl-CoA carboxylase	A4HJT6	LbrM.31.3340	LBRM2903_310043300	241 kDa	95 % (0.0053)
Lb14	N-terminal region of Chorein, a TM vesicle-mediated sorter, putative	A4H8K0	LbrM.16.0770	LBRM2903_160014400	604 kDa	95 % (0.049)
Lb15	Symplekin tight junction protein C terminal, putative	A4HJL9	LbrM.31.2640	LBRM2903_310035900	165 kDa	95 % (0.0028)
Lb16	replication factor C, subunit 3, putative	A4HMZ2	LbrM.34.3160	LBRM2903_340040300	40 kDa	95 % (0.011)
Lb17	hypothetical protein, conserved	A4HHV4	LbrM.30.0100	LBRM2903_300006000	24 kDa	95 % (0.042)
Lb18	hypothetical protein, conserved	A4HPR3	LbrM.35.4190	LBRM2903_350052100	51 kDa	95 % (0.0021)

**Bold** labelling refers to proteins specifically binding to *lncRNA4*5WT.

a Protein description based on TritrypDB annotation.

b Uniprot accession number.

c Gene identification in TritrypDB.

d Protein size in kilodalton (kDa).

e pValue and % of confidence obtained in the statistical analysis. One-Way ANOVA was used to compare S1m results of the negative control, the *lncRNA4*5WT and *lncRNA45*MUT pull-downs. Unpaired T-test was used to compare S1m results of the negative control with the *lncRNA45* pull-down. The differences were considered significant when p < 0.05.

Protein interaction network analysis was performed for the interactors of *lncRNA4*5WT (Fig. 5B) and *lncRNA45*MUT (Fig. 5C). A network consisting of six proteins was identified when the binding partners of *lncRNA4*5WT were analyzed (Fig. 5B). However, this network was disrupted when the interactors of *lncRNA45*MUT were imputed (Fig. 5C). Notably, two of the five proteins that exclusively bound to the native *lncRNA45* sequence (LbrM.07.0750 and LbrM.34.2420) are components of this network (Fig. 5A).

To identify the pathways *lncRNA4*5 might be involved in, the list of binding partners for *lncRNA4*5WT and *lncRNA45*MUT sequences was submitted to gene ontology (GO) enrichment analysis (Fig. 5D). Twelve and seventeen terms were retrieved for *lncRNA4*5WT and *lncRNA45*MUT, respectively. Eleven of these terms were shared between both protein lists. RNA processing (GO:0006396) was exclusively enriched in *lncRNA4*5WT, while six terms were exclusively enriched in *lncRNA45*MUT (Fig. 5D).

Since genome annotation can significantly impact GO analysis (56), we identified the orthologs of the *L. braziliensis* genes retrieved in the pulldown analysis within the *L. major* Friedlin genome, which has a more complete annotation to date, to compare the outcomes of this analysis (Supplementary Fig. 9). In this case, 13 and 20 terms were retrieved for *lncRNA4*5WT and *lncRNA45*MUT, respectively, with 12 terms shared between both protein lists. Consistently, RNA processing (GO:0006396) was exclusively enriched in *lncRNA4*5WT.

RNA/Protein immunoprecipitation (RIP:IP) was conducted to confirm *in vivo* relevance of *lncRNA45*-protein interactions identified in the S1m *in vitro* pulldown assay. The five proteins that interact only with *lncRNA4*5WT and not with *lncRNA45*MUT were tagged with HA at the N-terminus (Supplementary Fig. 10) and immunoprecipitated using anti-HA magnetic beads. The *L. braziliensis* M2903 parental cell line (untagged) was used as the negative control.

RNA that immunoprecipitated with the tagged candidate interacting proteins was extracted, rRNA-cleared and sequenced to confirm interaction with *lncRNA45* and identify other potential transcripts in the complex. Surprisingly, *lncRNA45* was enriched only in the eluate of the hypothetical protein (Lb4 - LBRM2903_280022100). A higher number of counts of *lncRNA45* transcript was also observed for XRN 5′-3′ exonuclease N-terminus (Lb2 - LBRM2903_160009500) in comparison to the negative control, though this difference was not statistically significant (Fig. 5E).

In the same assay, protein-protein interactions were investigated by mass spectrometry to evaluate potential networks involving *lncRNA45*. Interestingly, four out of the five analyzed proteins were found to interact with at least one of the 18 proteins identified as *lncRNA4*5WT-binding in the S1m pulldown assay (Fig. 5F), confirming some of the interactions predicted by STRING (*Search Tool for the Retrieval of Interacting Genes/Proteins*) (57).

To determine if these protein-protein interactions are RNA-dependent, a fraction of the eluate material was treated with RNAse and compared with the untreated eluate. Our findings show that the interaction of Lb1 (40S ribosomal protein) with Lb3 (Lupus La protein homolog) is lost after RNase treatment (Fig. 5G). The same is true for the interaction of Lb2 with Lb1, Lb3, Lb8, Lb11, and Lb12 (Fig. 5G). Three other protein-protein interactions (Lb5-Lb4, Lb5-Lb6, Lb2-Lb14) that were lost in RNase-treated compared to untreated samples were not significantly different from the negative control and may thus be artifacts (Fig. 5G). Notably, none of these proteins were detected in the negative controls, confirming their specific interaction with identified targets. This supports the significance of *lncRNA45* in regulatory protein complex interactions and functional cohesion.

## 3 Discussion

In this study, we used innovative *in silico* methodologies to classify putative ncRNAs of *Leishmania* based on the evolutionary conservation of their structure. Using reverse genetics, we identified a novel regulatory ncRNA, *lncRNA45*, with a conserved secondary structure. Our findings demonstrate that this lncRNA impact cellular fitness and that its structure is essential for its function.

Our applied computational approach integrated two strategies for genome-wide screenings of structured RNAs: simultaneous use of sequence and structural alignment along with evaluation of evolutionary conservation of secondary structure. To identify RNA families with shared structural features, we applied clustering methods based on conserved RNA structures. This structure-based RNA clustering conferred a rational basis for selecting ncRNAs for further investigation using reverse genetics. Among these, we analyzed the functional impact of a SNP that modifies the secondary structure of *lncRNA45*.

It is well established that gene expression modulation occurs throughout the parasite lifecycle and differences in RNA abundance between clinical isolates or under distinct environmental conditions have been demonstrated for *Leishmania* and other trypanosomatids [4,7,25]. How these parasites modulate gene expression in a context of polycistronic transcription and lack of canonical promoters, or the factors and mechanisms used to regulate gene expression are still not completely understood [4]. Only recently have studies began to suggest the existence of ncRNAs in trypanosomatids. Interestingly, computational and functional assays have demonstrated that among trypanosomatids only *Trypanosoma brucei* and *Leishmania Viannia* species possess a functional RNAi machinery [26]. But so far functional exploration and discovery of putative regulatory ncRNAs in *T. brucei* and *Leishmania* spp. is still incipient [12,27]. The recent identification of ncRNAs in trypanosomatids [8,9,[12], [13], [14]] introduced a new layer to gene expression regulation, complementing the more extensively characterized RNA-binding proteins [7,28]. Until recently, the biological and regulatory functions of ncRNAs in trypanosomatids remained unclear. However, a study published in 2022 [9], demonstrated that overexpression of the lncRNA *Grumpy* promotes *T. brucei* differentiation into stumpy forms, enhancing survival rates in infected mice.

While lncRNAs typically show limited sequence conservation across different organisms, specific structures regions within these transcripts—some even syntenically conserved—have been linked to both transcript stability and biological function [29]. To identify such conserved structured elements within trypanosomatid genomes, we performed a computational analysis at uncovering evolutionary conservation of RNA secondary structures. From this analysis, we selected one candidate, *lncRNA45*, for functional characterization. Here, we report, for the first time, both the *in silico* and experimental characterization of a structured lncRNA in *L. braziliensis*.

*LncRNA45* was identified in a comprehensive transcriptomic study aimed at deciphering gene expression regulation mechanisms in *L. braziliensis* [13]. This lncRNA was selected from among several candidates with a conserved secondary structure, because the RNA-seq analysis previously conducted indicated that it was differentially expressed throughout the lifecycle and is preferentially expressed in the intracellular, proliferative form that infects mammalian macrophages [13]. *LncRNA45* contains a structured motif (Locus 709), conserved in 6 *Leishmania* species (*L. braziliensis*, *L. panamensis*, *L. major* Friedlin, *L. donovani*, *L. infantum* and *L. amazonensis)* suggestive that this lncRNA structure may have a conserved essential function throughout *Leishmania*. In contrast, the full *lncRNA45* sequence is conserved only in four *Leishmania* species, all belonging to the *Viannia* subgenus.

The presence of *lncRNA45* in *L. braziliensis* was confirmed by multiple experimental approaches including, RNA-seq, RT-qPCR, RNA FISH, circularization assay and northern blotting, confirming that this transcript is consistently expressed and not an artifact of the RNA-seq data [13]. Characterization of *lncRNA45* shows it does not undergo the same processing routes as mRNAs in *L. braziliensis*, a hallmark feature of lncRNAs in diverse organisms [30], and one that appears to be shared by ncRNAs in *Leishmania* as well [8]. *LncRNA45* has a short (17 nt) poly (A) tail but lacks a 5′ spliced-leader sequence, a characteristic of mature mRNAs in *Leishmania*. Treatment with a decapping enzyme (TAP) suggests that the 5′end of *lncRNA45* is protected and that the transcript is not monophosphorylated. Further studies must be conducted to elucidate whether *lncRNA45* undergoes alternative capping and to characterize it biogenesis, maturation and stabilization mechanisms, factors that are likely critical for its protection from degradation and its functional activity in the parasite [31].

Loss-of-function analysis showed that *lncRNA45* plays a direct role in parasite growth in culture, consistent with its increased expression during this life stage. Interestingly, the reduced cell density observed in the knockout line, compared to the parental strain was not due to an increased doubling time. This suggests that the growth defect may stem from an increased death rate or disruption of a signaling pathway, potentially affecting the parasite's quorum sensing capacity. The hypothesis that these mutants might be more susceptible to nutritional stress was ruled out, as no differences in their ability to recover from nutritional stress were observed.

To determine whether this biological role is structure-dependent, we used *in silico* predictions to design a single nucleotide substitution in Locus 709 (C50G) which was expected to disrupt its secondary structure. The inability of the modified *lncRNA45* (*lncRNA45*MUT) to rescue the growth phenotype of the knockout background—unlike the wild-type sequence (*lncRNA4*5WT)—confirmed that the structural integrity of Locus 709 is essential for *lncRNA45* function. This observation is consistent with findings from other structured lncRNAs, such as *lnc-31* and *Braveheart*, where point mutations compromise critical molecular interactions, including proteins or RNA scaffolding, thereby impairing their function [16,32].

The functional mechanism in which a lncRNA is involved is closely related to its subcellular localization [10]. Cytoplasmic lncRNAs are typically involved in regulating mRNA stability, RNA processing, translation rates or protein function, whereas nuclear lncRNAs act by modulating transcriptional activity [33]. RNA FISH revealed that *lncRNA45* was mostly observed in the cytoplasm, without a preferential subcellular distribution, being spotted in different regions of the cytoplasm of *L. braziliensis*. Additionally, RNA-seq analysis indicated that *lncRNA45* does not modulate the expression of specific mRNAs in this parasite*.* Based on these findings, we hypothesize that *lncRNA4*5 might influence RNA processing, or translation efficiency; directly interacting with proteins to modulate their activity or stability. Similar mechanisms were described for *lnc-31* which positively regulate Rock1 protein levels, without affecting its mRNA levels, suggesting a potential positive role of *lnc-31* in promoting translation of Rock1 mRNA [32].

*In vitro* pulldown assays using both *lncRNA4*5WT and *lncRNA45*MUT sequences as bait confirmed the importance of the secondary structure formed by Locus 709 for *lncRNA45* function. The presence of the C50G substitution disrupted the binding of five proteins to this lncRNA, underscoring the structural dependency of these interactions. The identity of the proteins pulled down with *lncRNA45* suggests this transcript may be involved in regulating RNA processing or cell-cell signaling pathways, particularly quorum sensing, which corroborates the reduced parasite density phenotype observed in loss-of-function assays.

Although lncRNA-protein interactions were not statistically confirmed for all five proteins in RIP assays, our RNA-seq data revealed higher *lncRNA45* read counts in the eluates compared to the negative control for XRN 5′-3′ exonuclease (Lb2) and for two biological replicates of nucleolar protein Nop53 (Lb5). For the hypothetical protein (Lb4), *lncRNA45* was significantly enriched in the eluate. It is important to note that *lncRNA45* is not an abundant transcript in *L. braziliensis* M2903, as observed in the parental cell line RNA-seq data. This low abundance may have affected the sensitivity of the statistical analysis, as this limitation was not observed for other more abundant lncRNAs investigated in the same system. Interestingly, the interaction between Lb4 and Lb5 proteins was shown to be RNA-dependent as was the interaction of Lb2 with six additional proteins (Lb1, Lb3, Lb8, Lb11, Lb12 and Lb14). These findings raise the possibility that *lncRNA45* may play a scaffolding role, stabilizing the protein complex via RNA-mediated interactions.

An in-depth literature review of the five proteins shown to interact with *lncRNA45* in a structure-dependent manner revealed that at least one of them, the Lupus La homolog, has previously been identified as an interactor of two other long non-coding RNA: XIST in mouse and Grumpy in *T. brucei*. Notably, both Lupus La homolog and the XRN 5′-3′ exonuclease have been implicated in regulating *T. brucei* growth in loss-of-functions studies demonstrating their impact on parasite growth [34], mirroring the phenotype observed in *L. braziliensis lncRNA45* knockout parasites.

Interestingly, three of these proteins (40S ribosomal protein S9, Nop53 and Lupus La) have also been associated with ribosome biogenesis and translation initiation [[35], [36], [37]], supporting the hypothesis that *lncRNA45* may be involved in post-transcriptional regulation. RIP-seq MS data not only confirmed these interactions but also expanded *lncRNA45*-protein network to include 18 putative partners. Importantly, interactions between the Lupus La homolog interaction a both the 40S ribosomal protein and Nop53 were disrupted upon RNAse-treatment of RIP samples, suggesting that *lncRNA45* and/or other RNAs are required to mediate or stabilize these protein-protein interactions.

To our knowledge, this is the first demonstration of a secondary structure-dependent lncRNA activity in trypanosomatids, and the first functional characterization of a regulatory ncRNA in *Leishmania*. This study encourages the search for other conserved, structured regulatory lncRNAs that could serve as potential therapeutic targets or vaccine candidates.

In conclusion, our study underscores the relevance of long non-coding RNAs (lncRNAs) in the regulation of gene expression in trypanosomatids. Through the characterization of *lncRNA45* in *Leishmania braziliensis*, we demonstrated that the conserved secondary structure in this lncRNA is essential for both its interaction with proteins and biological function. Loss-of-function analyses revealed that *lncRNA45* plays a crucial role in promastigote growth, highlighting the importance of its secondary structure for its regulatory role.

The protein interaction network identified via pulldown assays and RIP-seq implicates *lncRNA45* in RNA processing and cell signaling pathways, notably quorum sensing. Moreover, the interaction of *lncRNA45* with proteins as Lupus La homolog, 40S ribosomal protein S9, and Nop53, previously linked to ribosome biogenesis and translation initiation, further supports its involvement in post-transcriptional regulation.

Altogether, our findings provide compelling evidence for the functional relevance of regulatory ncRNAs in *Leishmania* and demonstrate, for the first time, a lncRNA whose activity is dependent on the secondary structure in trypanosomatids. This study contributes to a deeper understanding of the complexity of RNA-based regulation in these parasites and reinforces the potential of structured lncRNAs as novel molecular targets in the fight against leishmaniasis.

## 4 Materials and methods

### 4.1 Quality analysis of trypanosomatids genome

Genomic assembly completeness was assessed using BUSCO v4.0.0 (Benchmarking Universal Single-Copy Orthologs) [38]. BUSCO identifies candidate regions via local alignment against amino acid consensus sequences [39], extracts gene models using block profiles [40], and evaluates them against profile Hidden Markov Models (HMMs) to quantify genome completeness.

BUSCO tool suite (v.4.0.0) metrics were used in genome mode with the *euglenozoan_odb10* dataset (creation date: 2019-11-21; 31 species; 130 universal orthologs) to assess the quality and completeness of the assemblies of Trypanosomatids genomes. The analysis was performed in *genome mode*, and *Leishmania tarentolae* was selected as the AUGUSTUS parameter, as it is the only *Trypanosomatid* species with an available training annotation file.

### 4.2 Genome alignment and search for conserved regions

Lastz (v1.04.03) [18] was used to align ten trypanosomatid genomes to *Leishmania braziliensis* MHOM/BR/75/M2903, covering 99 % of its genome. After filtering for positive scores, ≥3 sequences per alignment, and a minimum alignment size of 20, coverage was reduced to 92 %. Alignment windows of 40–120 nt (step size: 40, ≥3 sequences) further filtered coverage to 77 %. Overlapping lncRNA annotations yielded 12,969 windows, analyzed by RNAz on both strands. False-discovery rates were estimated by shuffling windows 100 × with SISSIz [41] while preserving di-nucleotide content.

### 4.3 Predictions of mutations that affect the RNA structure

*RNAsnp* tool with parameters Model1 p.value 0.01, Model2 p.value 0.01, winsize 200, winsize extension 200 [42,43] were used to predict point mutations that can affect the stability of RNA secondary structures.

### 4.4 Cell lines culture

*L. braziliensis* M2903 expressing Cas9 and T7 RNA polymerase from pTB007 plasmid, and tdTomato in the SSU locus was used [44,45]. Promastigotes were cultured at 25 °C in M199 medium supplemented with 2.2 g/L NaHCO_3_, 0.1 mM adenine hemi sulfate, 0.005 % haemin, 40 mM HEPES pH 7.4, 100 μg/mL penicillin/streptomycin (Pen/Strep), 1 μM Biopterin and 10 % heat-inactivated FBS 10 %. The selection drugs were added at 32 μg/mL hygromycin B (Gibco™), 50 μg/mL Nourseothricin (Sigma-Aldrich®), and 20 μg/mL puromycin dihydrochloride (Gibco™).

Axenic amastigotes were obtained by incubating metacyclic promastigotes in 100 % FBS at 33 °C with 5 % CO_2_. THP-1 cells for infectivity assays were maintained in RPMI + 10 % FBS at 37 °C with 5 % CO_2_, passaged every 3 days or when cell density was close to 10^6^ cell/mL.

### 4.5 DNA extraction

*L. braziliensis* total DNA extraction for cloning and sequencing purposes was done using DNeasy Blood & Tissue Kits (Qiagen) according to the manufacturer's specifications. For colony screening the protocol described by Rotureau, Gego and Carme [46] was employed.

### 4.6 RNA extraction

Total RNA was extracted from *L. braziliensis* cultures using the Directzol kit Quick-RNA Miniprep (Zymo Research) according to the manufacturer's instructions. After extraction, RNA samples were treated with TURBO™ DNAse to remove any DNA remains.

### 4.7 Real-time quantitative PCR (RT-qPCR)

Differential expression was assessed by RT-qPCR using primers specific for *lncRNA45* (*lncRNA45*_RT-F and *lncRNA45*_RT-R). The 7SL RNA gene served as a normalizer, amplified with primers 7SL_RT-F and 7SL_RT-R (Supplementary File 1). RT-qPCR was performed using SYBR Green/ROX qPCR MasterMix, on a StepOnePlus™ Real-Time PCR System (95 °C for 10 min, followed by 40 cycles of 95 °C for 15 s, 60 °C for 60 s, and 72 °C for 20 s). Ct values were normalized to 7SL RNA, and differential expression was calculated relative to the procyclic promastigote stage using the 2ΔΔCt method [47].

### 4.8 RNA circularization

The RNA circularization assay was performed as described by Hang, Deng, Liu, Mo and Cao [24], with modifications. Total RNA (5 μg) from *L. braziliensis* M2903 was either treated (TAP+) or not (TAP−) with Tobacco Acid Pyrophosphatase (FirstChoice® RLM-RACE - Invitrogen™). T4 RNA ligase (NEB), ATP, and RNasin® (Promega) were added for circularization. The resulting circular RNA (cRNA) was precipitated and reverse transcribed using *lncRNA45*_cRT primer and TransScript® II First-Strand cDNA Synthesis SuperMix kit (TransGen Biotech). PCR amplification was conducted using Platinum™ Taq DNA Polymerase High Fidelity (Invitrogen™) and the primers *lncRNA45*_cF1 and *lncRNA45*_cR1 followed by a nested PCR using primers *lncRNA45*_cF2 and *lncRNA45*_cR2 primers (Supplementary File 1). PCR products were cloned into pGEM®-T Easy Vector (Promega) for transformation in *E. coli* DH5-α. Positive clones were confirmed by PCR with M13 primers and analyzed by Sanger sequencing. Sequences were mapped to the *L. braziliensis* M2903 genome using Geneious Software.

### 4.9 Northern blotting

Total RNA was extracted from log-phase parasites using the mirVana™ Total RNA Isolation Kit (ThermoFisher Scientific) and treated with Turbo DNase. 4 μg of total RNA was mixed with glyoxal mix, denatured at 50 °C for 40 min and electrophoresed on a 2 % agarose gel stained with SYBR Green II. The gel was imaged on an iBright imager (Invitrogen™) after running at 150 V for 90 min. RNA was transferred to a Hybond®-N+ membrane by capillarity in SSC buffer overnight, UV cross-linked (20,000 μJ/cm^2^, 1 min), and dried at 80 °C for 1 h. For radioactive probing, a PCR-generated amplicon from the ncRNA target cloned into pJET II was labeled using the DIG Northern Starter Kit (Sigma). Hybridization occurred at 42 °C for 18 h in a buffer containing tetrasodium pyrophosphate, SSC, Denhardt's solution, SDS, and heparin, followed by washes with 0.1 % SDS in 2× SSC. The membrane was exposed to photographic film at −80 °C for seven days, developed, and imaged with a conventional camera.

### 4.10 RNA FISH

Approximately 1 × 10^7^ cells were centrifuged at 2000×*g* for 10 min, washed once with PBS and resuspended in a solution of 4 % formaldehyde in PBS. Cells were spread in a 0.01 % poly-lysine-coated slide (Sigma-Aldrich®) for 30 min and washed once with PBS. Hybridization of Type I probes, labeled with Alexa Fluor 546 fluorochrome (Thermo Fisher™) was conducted using ViewRNA™ ISH Cell Assay Kit (Invitrogen™) according to the manufacturer instructions and the protocol described by Kramer [48]. Cells were imaged in a LSM 780/LSM 7 MP Carl Zeiss multiphoton microscope with 0.75 magnification.

### 4.11 Generation of transfectant line

*Transfections*. *L. braziliensis* M2903 Cas9/T7 promastigotes were transfected using Amaxa™ Nucleofactor™ (Lonza) X-001 program in Tb-BSF 1× buffer. After 16 h in M199 medium, cells were cloned on M199-agar plates with biopterin (1 μM) and selection drug. Genomic modifications were confirmed by PCR.

*Knockout* (*ΔlncRNA45*). A *lncRNA45* knockout (*ΔlncRNA45*) line was generated using CRISPR/Cas9, following the method from Ref. [44]. sgRNAs were designed using EuPaGDT [49], targeting sequences near the 5′ and 3′ ends of *lncRNA45*. Homology arms (30 nt) flanking the Cas9 cleavage site were included in the donor DNA. Transfected clones were screened by PCR using primers Conf-F and Conf-R. Homozygous knockouts showed only the donor DNA band, while heterozygous clones displayed both *lncRNA45* and donor DNA bands.

*Overexpression* (OE-*lncRNA45*). For overexpression, *lncRNA45* was amplified from *L. braziliensis* M2903 genomic DNA using OE_*lncRNA45*_*Not*I-F and OE_*lncRNA45*_*Bam*HI-R primers. The fragment was cloned into pSSU-Sat to generate pSSU-*OElncRNA45*-Sat. Transfected clones were screened by PCR using *lncRNA45*-F and Sat-R primers. A control plasmid (pSSU-GFP-Sat) was verified using GFP-F and Sat-R primers.

*Add-back*. Wild-type (*lncRNA4*5WT) and mutant (*lncRNA45MUT*) sequences were synthesized and amplified using *lncRNA45*_*Not*I-F and *lncRNA45*_*Bam*HI-R. The fragments were cloned into pSSU-Sat, generating pSSU-*lncRNA4**5*WT-Sat and pSSU-*lncRNA45MUT*-Sat, and transfected into *ΔlncRNA45* promastigotes. Verification was done by PCR using *lncRNA45*-F and Sat-R, while pSSU-GFP-Sat was used as a mock control.

*HA-tagged cell line*. Target proteins were HA-tagged using CRISPR/Cas9 as described in Refs. [44,50]. sgRNA and donor DNA primers were designed using LeishGEdit, which also provided primers for confirmation. The complete list of primers used in this study is provided in Supplementary File 1.

### 4.12 Phenotypic assays

*Growth Curve*. Parental and transfectant cell lines' growth rates were compared using AUC analysis over a 7-day growth curve. Cultures of 2 × 10^5^ promastigotes/mL in M199 medium at 25 °C were counted daily for 7 days. Biological triplicates were analyzed using GraphPad Prism 8.0.2. Significance was assessed via non-parametric *t*-test (two groups) or One-Way ANOVA with Tukey's test (multiple groups).

*Doubling Time*. Doubling time (DT) was measured by adjusting culture density to 1 × 10^6^ promastigotes/mL in M199, incubating at 25 °C, and counting parasites daily for 4 days. DT was calculated at the end.

*Nutritional Stress*. Recovery from nutritional stress was assessed by incubating 1 × 10^6^ promastigotes in M199 (unstressed) or PBS (stressed) for 4 h. After centrifugation, fresh M199 with MTT was added, and after 24 h, formazan crystals were dissolved with DMSO. Absorbance at 570 nm was measured to calculate cell viability. Experiments were performed in triplicate with five technical replicates. Data were analyzed using One-Way ANOVA and Tukey's test.

*Metacyclogenesis*. Promastigotes were cultured for 5 days at 25 °C, centrifuged, and resuspended in M199. A Ficoll gradient (20 %, 10 %) was used to isolate metacyclic promastigotes. The percentage of metacyclogenesis was calculated and compared to the parental line using a non-parametric *t*-test.

*THP-1 Infection*. THP-1 monocytes were differentiated into macrophages using PMA and infected with metacyclic promastigotes (10:1 ratio). Infection rates and amastigotes per macrophage were measured at 24 h and 48 h using the ImageXpress Micro XLS System. Data were analyzed using One-Way ANOVA and Tukey's test.

*Oxidative Stress*. Axenic amastigotes were cultured in M199 pH 5.4 at 33 °C for 72 h, then exposed to 300 mM H_2_O_2_ (stressed) or no H_2_O_2_ (non-stressed) for 24 h. MTT was added, and after 24 h, formazan crystals were dissolved with DMSO. Absorbance at 570 nm was measured to calculate viability. Experiments were performed in triplicate with five technical replicates. Data were analyzed using One-Way ANOVA and Tukey's test.

### 4.13 S1m-mediated RNA pull-down

*LncRNA45* (native/mutated) was amplified, digested (PacI, NcoI), and cloned into pUC57-T7-4xS1m. RNA was transcribed *in vitro* (MEGAscript™ T7), immobilized on streptavidin-coated magnetic beads (SA-beads), and incubated overnight with *L. braziliensis M2903* protein lysate, pre-cleared with SA-beads at 4 °C. After washing, bound proteins were eluted, boiled in Laemmli buffer, and analyzed by SDS-PAGE. Protein digestion and MS analyses were performed at CHU de Québec. Gel bands were excised, processed, and peptides analyzed by nano-LC-MS/MS (Dionex UltiMate 3000-Orbitrap Fusion). MS data were acquired in data-dependent mode (XCalibur 4.3, 120,000 resolution, 350–1800 *m*/*z*). MS/MS spectra were searched against the *L. braziliensis* Uniprot database (8153 entries) using Mascot 2.5.1, with fixed carbamidomethylation and variable modifications (deamidation, oxidation, methylation/dimethylation). Identifications were validated in Scaffold 5.0.1 (FDR <1 %, ≥2 peptides). Statistical significance was assessed using One-Way ANOVA (p < 0.05).

### 4.14 Protein/RNA immunoprecipitation (RIP)

Immunoprecipitations were conducted as described previously by Walrad et al. [35,36] with modifications. *L. braziliensis* promastigotes (1 × 10^9^) were lysed in IP-Lysis Buffer, frozen, sonicated, and centrifuged. Supernatant was incubated with 1 mg of Pierce™ Anti-HA Magnetic Beads (Thermo Scientific™) for 2 h at 4 °C. Negative control beads were blocked with HA peptide. Beads were washed, split, and processed for RNA extraction, protein elution (Laemmli buffer, 95 °C), or RNase treatment. Immunoblotting was performed with anti-HA (1:5000) and anti-rabbit IgG (1:10,000) antibodies, detected using Amersham ECL (Cytiva) on a BioRad ChemiDoc.

*Mass Spectrometry Analysis of RIP Samples*. SDS-PAGE-separated samples were in-gel digested with trypsin, peptides extracted, and loaded onto EvoSep C18 columns for separation (100 SPD gradient, EvoSep One). MS analysis used timsTOF HT (Bruker) with PASEF-DIA acquisition. Data were analyzed with DIA-NN (1 % FDR) against *L. braziliensis* TriTrypDB, and quantification was performed using Quant-UMS and KINME (q < 0.01, ≥2 peptides). Differential abundance was assessed with Limma in FragPipe-Analyst (adjusted p < 0.05).

*RNA-seq.* RNA libraries were prepared (Macrogen, Inc., Illumina Ribo-Zero & TruSeq) and sequenced (∼40M 101 bp paired-end reads). Reads were mapped with *bowtie2* (TriTrypDB v66) and counted using *htseq-count*. Differential expression analysis was performed with DESeq2 (*p* < 0.05, fold-change >1.5).

### 4.15 GO analysis

The list of accession numbers was converted into Gene ID on the Uniprot website. A search for the list of IDs was conducted on the TriTrypDB website, and the results were sent to gene ontology enrichment of biological processes. A p-value of 0.05 was used as the cutoff for significance. The retrieved terms, fold-change enrichment, number of genes in the term and p-value were used to generate a heat map using GraphPad Prism 10.

### 4.16 Interactions networks

Interactions networks for the proteins identified in the pull-down assay for both *lncRNA4*5WT (18 proteins) and *lncRNA45*MUT (13 proteins) were retrieved from the STRING database [46] using all available evidence types. Cytoscape [51] was used to highlight proteins and interactions shared between the networks. An interactive version of the networks was submitted to the NDEx database [52].

## CRediT authorship contribution statement

**Caroline R. Espada:** Writing – review & editing, Writing – original draft, Methodology, Investigation, Formal analysis, Data curation, Conceptualization. **Christian Anthon:** Writing – review & editing, Writing – original draft, Methodology, Formal analysis, Data curation, Conceptualization. **Rubens D.M. Magalhães:** Writing – review & editing, Formal analysis, Data curation, Conceptualization. **José Carlos Quilles Junior:** Writing – review & editing, Methodology, Investigation, Data curation. **Natalia M.M. Teles:** Writing – review & editing, Investigation. **Fabiano S. Pais:** Writing – review & editing, Methodology, Formal analysis. **Lissur A. Orsine:** Writing – review & editing, Methodology, Investigation, Formal analysis, Data curation. **Letícia de Almeida:** Writing – review & editing, Formal analysis, Data curation. **Tânia P.A. Defina:** Writing – review & editing, Methodology. **Adam Dowle:** Writing – review & editing, Methodology, Formal analysis, Data curation. **Jan Gorodkin:** Writing – review & editing, Supervision, Formal analysis, Conceptualization. **Pegine B. Walrad:** Supervision, Funding acquisition, Formal analysis, Conceptualization. **Angela K. Cruz:** Writing – review & editing, Writing – original draft, Supervision, Resources, Project administration, Funding acquisition, Conceptualization.

## Funding

This study was funded by 10.13039/501100001807FAPESP (grants 2018/14398-0, 2015/13618-8), 10.13039/501100003593CNPq (305775/2013-8, 152584/2022-6), and 10.13039/501100002322CAPES (Finance Code 001) to A.K.C. Fellowships were awarded by 10.13039/501100001807FAPESP to C.R.E. (2020/00087-2 and 2022/10270-4), R.D.M.M. (2019/18607-5) J.C.Q.J. (2020/00088-9, 2021/15182-3), L.A. (2017/19040-3), and L.A.O. (2021/10043-5, 2023/18057-0) and by the Medical Research Council to N.M.M.T. and P.B.W. InTEGRL (MR/V031511/1). Funding agencies had no role in study design, data collection, analysis, or publication.

## Declaration of competing interest

The authors declare that they have no known competing financial interests or personal relationships that could have appeared to influence the work reported in this paper.
